# Dual-Routes and the Cost of Determining Least-Costs

**DOI:** 10.3389/fpsyg.2017.01943

**Published:** 2017-11-07

**Authors:** Steven Phillips, Yuji Takeda, Fumie Sugimoto

**Affiliations:** ^1^Mathematical Neuroinformatics Group, Human Informatics Research Institute, National Institute of Advanced Industrial Science and Technology, Tsukuba, Japan; ^2^Automotive Human Factors Research Center, National Institute of Advanced Industrial Science and Technology, Tsukuba, Japan

**Keywords:** dual-route, least-cost, feature search, conjunction search, systematicity, universal construction, category theory

## Abstract

Theories of cognition that posit complementary dual-route processes afford better fits to the data when each route explains a part of the data not explained by the other route. However, such theories must also explain why each route is invoked, lest one can fit any data set with enough alternatives. One possible explanation is that route selection is based on a least-cost principle: the route that requires fewer cognitive resources (including time) relative to the goal at hand. We investigated this explanation with a dual-display version of visual search, where the target could be identified via opposing (easy or hard forms of) feature and conjunction search conditions. The data support a contextualized version of the least-cost principle in that the cost of computing least-cost also influences route selection: participants assessed alternatives, but only when the cost of that assessment was relatively low.

## 1. Introduction

Cognitive science is replete with competing theories on the nature of cognitive representations and processes, for example, imaginal vs. propositional (Thomas, 2016), associative vs. relational (Halford et al., 2014), probabilistic vs. rational (Chater and Oaksford, 2008; Johnson-Laird et al., 2015), recursive vs. non-recursive (Watumull et al., 2014; Everett, 2016), and so on. Empirical evidence is sought in support of one alternative over the other. Yet, such support is rarely one-sided, which makes adjudicating between competing theories difficult.

One possible reason for the apparent indeterminacy is that cognition may be pluralistic in the kinds of processes that are deployable to complete a task. This possibility is given voice as the distinction between two kinds of cognitive processes (see, e.g., Kahneman, 2011; Evans and Stanovich, 2013), which are sometimes called *System 1* and *System 2*. Characteristically, System 1 processes are fast, domain-specific, and resilient to interference from concurrent task demands, whereas System 2 processes are slow, domain-general, and affected by concurrent demands, such as working memory load. So, for example, a cognitive system may trade off understanding for rapid response by deploying a “quick-fix” solution in response to a change in circumstances (context).

Pluralistic (or, hybrid) theories have clear benefits and costs when compared to monolithic theories, in general. The main benefit is a better fit to the extant data when the component theories are complementary: each component explains a part of the data that is not explained by the other component. The cost, however, is that hybrid theories are held to a higher explanatory standard: not only must each component theory explain some part of the data, but the hybrid theory is also required to explain *why* the split is that way, i.e., other than just by appeal to fitting the data (Aizawa, 2003).

Suppose, for example, a hybrid associative-symbolic theory to account for those aspects of cognition with the *systematicity* property (Fodor and Pylyshyn, 1988; Fodor and McLaughlin, 1990) and those aspects where systematicity is not present (Niklasson and van Gelder, 1994; Johnson, 2004). The symbol system component is invoked to account for the systematicity property: e.g., having the capacity to understand the proposition that *John loves Mary* implies having the capacity to understand the structurally-related proposition that *Mary loves John*, because there is a symbolic representation for the common semantic relation *loves*. The associative component is invoked to account for idioms, e.g., *John kicked the bucket* (i.e., *John died*), which is not structurally related to the superficially similar *John kicked the ball*, as an associative link from the proposition to its idiosyncratic meaning. These two instances of *kick* are not systematically related, and the association component accounts for this fact. The question then arises as to why and under what conditions does the system interpret the proposition idiomatically or structurally (as in *John kicked the ball*). For specific instances, one can appeal to historical context (e.g., John had just died). However, a hybrid theory is required to spell out the general causal basis upon which such possible alternatives are determined (see Aizawa, 2003).

In regard to systematicity vs. non-systematicity properties, one hypothesis pertains to a cost/benefit tradeoff between systematic and non-systematic (associative) processes (Phillips et al., 2016). The basic idea is that systematicity is a consequence of a kind of *universal* construction (Phillips and Wilson, 2010) that in certain circumstances requires more resources than associative processes, which do not have the systematicity property. Failure to exhibit systematicity occurs when the association route is deployed, due to its cost advantage in some contexts. Support for this hypothesis was observed in a task that involved learning stimulus-to-stimulus mappings generated from an underlying rule. When the number of mappings to be learned was small, participants learned the task without inducing the rule—no systematicity (association route). However, evidence for induction was observed when the task involved larger numbers of mappings—systematicity (universal construction route). These results suggested a pluralistic system with alternative routes selected on the basis of least cost contextualized by task conditions (Phillips et al., 2016).

### 1.1. Overview: motivation and design

The purpose of the current study is to examine this (contextualized) least-cost suggestion directly by explicitly varying the cost of two alternative ways of completing a visual search task: find a target object among non-targets. *Prima facie*, visual search seems far from a general notion dual-route in cognition. Although psychologists have long regarded visual attention in terms of bottom-up vs. top-down mechanisms (Treisman and Gelade, 1980), visual search processes appear to have little to do with reasoning. However, there are several underlying considerations that conjointly motivate visual search as a paradigm for studying dual-route cognition. Firstly, visual search for targets defined by conjunctions of features (e.g., color and shape) reportedly evoke greater prefrontal activity than targets defined by a single feature (Buschman and Miller, 2007; Phillips et al., 2012). Secondly, prefrontal cortex is differentially active with changing (relational) complexity of reasoning tasks (Waltz et al., 1999; Christoff et al., 2001). Thirdly, a capacity to process conjunctions of visual features and some more complex forms of reasoning appear around the common age of five (Lloyd et al., 2009; Halford et al., 2014). And, fourthly, underlying these differences in cognitive capacity is a common *category theory* (Mac Lane, 1998) notion of a (categorical) product (Phillips et al., 2009, 2012), which we have also employed in the context of systematicity (Phillips and Wilson, 2010). Data and theory point to a common connection, when taken together. On a pragmatic level, (feature/conjunction) visual search is a well-established paradigm that yields robust and reproducible results. Hence, the general motivation for employing a visual search paradigm is to manipulate conditions so as to effectively exercise such differences, e.g., products vs. non-products as dual-routes, see section 5.

Visual search involves identifying a target object in a field filled with non-targets (distractors). Search is typically faster (low cost) when the target is uniquely identifiable along a single dimension (e.g., color)—*feature* search, and slower (high cost) when the target shares more features with distractors—*conjunction* search (Treisman and Gelade, 1980). However, search difficulty is also affected by other factors, such as feature-feature similarity (or, discriminability). Thus, visual search affords a variety of ways to manipulate cost. A pilot study employed a “dual-display” variation of a visual search paradigm (Wolfe et al., 1989) to test the basic least-cost assumption. Participants were presented with two search fields in opposing (left/right) sides of the computer monitor that was used for stimulus presentation. Each field contained the same target, but different distractors, so that one field specified feature search and the other specified conjunction search. Response times and electro-oculogram (EOG) measures of eye movements revealed that participants preferred to search for the target in the feature field, thus establishing a clear preference for feature search as the least-cost alternative in this context. For the current study, we examine whether this preference is absolute or contextualized by other cost factors by manipulating the difficulty (hence, cost) of feature and conjunction search independently (Throughout this study, we consider cost in terms of response time, because response accuracy was near ceiling and did not significantly differ across all conditions).

Feature and conjunction search difficulty/cost can be manipulated independently by varying feature discriminability in the former and dimensionality in the latter. For feature search that means manipulating saliency: e.g., search of a red target among green vs. light red distractors. For conjunction search that means manipulating the number of conjoined features that uniquely identify the target: e.g., color and shape vs. color and shape and size, while keeping feature discriminability constant.

The experiment that follows is designed to assess the (potentially) contextualized nature of the least-cost principle. Three competing hypotheses arise in this regard.

1. The *independent* hypothesis simply says that choice of alternative routes is independent of task conditions.
2. The *absolute* least-cost hypothesis says that choice is based on the (absolute) cost of each route, which is determined when that route is the only option.
3. The *context* least-cost hypothesis says that choice is contextualized by other factors.

Behavior and eye movement data provided support for the third hypothesis, motivating a refinement called the *cost of computing least-cost* hypothesis and a follow-up experimental test (section 4). This refinement is motivated by a common-sense intuition: one is unlikely to spend $10 in search of a bargain that saves at most five. Accordingly, we hypothesized that the cost of determining the least-cost alternative also influences the choice of search field. The idea is that a cognitive system defaults to a less accurate, but less costly determination when the cost of determining least-cost is itself comparable to the cost of completing a task via either alternative.

Note that there are two senses of dual used throughout this work. One sense, termed *dual-display*, refers to the task design in the form of two search displays from which participants can identify the target, which is the same item in both displays (for a given trial). The other sense, termed *dual-route*, refers to putative cognitive processes (e.g., bottom-up vs. top-down attention). The relationship between these two senses is discussed in section 5.

## 2. Methods

Participants performed a visual search task: search for a target object in a display that also contained non-target (distractor) objects. Each object in the display field was a small or large colored shape that had a notch at the top or bottom of the object. The target of search was a large red ring, and the objective was to determine whether the notch was located at the top or bottom of the target. For test trials, each search display contained two sets of objects that consisted of a target and 15 distractors, totalling 16 objects for each set. One set was displayed in the left hemifield, and the other set was displayed in the right hemifield. Notch location for the target in each set was the same: either at the top of both targets, or at the bottom of both targets. So, on each test trial, participants could determine notch location by searching only in the left hemifield, or only in the right hemifield. For control trials, the search display contained only one set of objects (one target and 15 distractors) displayed either only in the left hemifield, or only in the right hemifield.

### 2.1. Participants

Twenty-three adults (19–34 years of age, 14 males, 20 right handed) participated in the experiment and were paid for their time. All had normal or corrected-to-normal eyesight. This study was carried out in accordance with the recommendations of “Guidelines for handling ergonomic experiments, Committee on Ergonomic Experiments, Bioethics and Biosafety Management Office, Safety Management Division, National Institute of Advanced Industrial Science and Technology” and approved by the Committee, with written informed consent from all participants. All participants gave written informed consent in accordance with the Declaration of Helsinki.

### 2.2. Apparatus and stimuli

**Figure 1:**
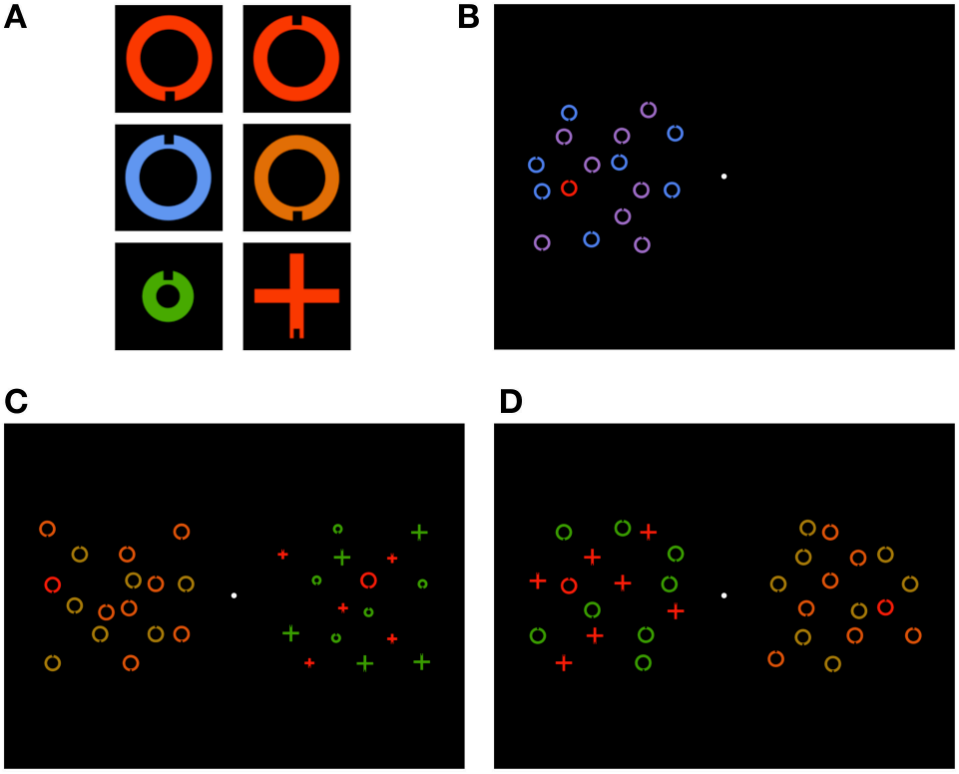
Example **(A)** targets with a notch located at the bottom and top of ring (top row), feature-low and feature-high distractors (middle row) and conjunction-low and conjunction-high distractors (bottom row), **(B)** control trial in the feature-low condition, with display objects in the left hemifield, **(C)** test trial in the high-low condition with feature and conjunction search in the left and right hemifields, respectively, and **(D)** test trial in the high-high condition with feature and conjunction search in the right and left hemifields, respectively.

Stimuli were presented by a notebook computer with a 43 cm (width) and 33 cm (height) external display—screen resolution was 1, 920 × 1, 200 pixels and refresh rate was 60 Hz—that was placed about 57 cm from the participant, so that 1 cm was approximately 1° field of view. The stimuli were one or two sets of stimulus objects that were identifiable by shape (ring, or cross), color (red, blue, green, purple, orange, or beige) and size (small, or large) features. Each set was arranged in an imaginary 6 × 6 grid of cells. The grids were centered on the horizontal midline, and equidistant from the vertical midline, with a 3.5 cm separation. The cells were 2.4 cm length squares. Each object was placed at the center of a cell with random jitter of 0–0.54 cm. Small and large objects were 0.89 and 1.44 cm (width/height). Stimuli were displayed on a black background. Luminance was 42–44 cd/m^2^. Examples are shown in Figure 1.

Behavioral data were acquired via the keyboard. EOG was recorded with a digital amplifier (Brain Products, QuickAmp). Ag/AgCl electrodes were placed above and below the left eye (vertical EOG) and at outer left and right canthi (horizontal EOG). Electrode impedance was kept below 10 kΩ. EOG signals were amplified with a high-cutoff of 200 Hz and digitized at a sampling rate of 1,000 Hz.

### 2.3. Conditions

For test trials, the experiment involved a 2 × 2 factorial design. The factors were *feature search cost* (low, high) and *conjunction search cost* (low, high).

- *Feature search*: In the feature search conditions, the target was uniquely identifiable by the color feature, i.e., all distractors were large non-red rings. In the low cost condition, about half (seven, or eight) of the distractors were blue and the other distractors were purple. In the high cost condition, about half of the distractors were orange and the other distractors were beige.
- *Conjunction search*: In the conjunction search conditions, the target was uniquely identifiable by any two-way conjunction (low cost), or only the color-shape conjunction (high cost). In the low cost condition, a distractor was either a small green ring, a large green cross, or a small red cross. There were five distractors for each of the three feature combinations. In the high cost condition, about half (seven, or eight) of the distractors were large green rings, and the other distractors were large red crosses.

The feature-conjunction search test conditions were: low-low, low-high, high-low, and high-high.

For control, there was only one search display set for each control trial: either a feature or conjunction search display at either low or high cost. Accordingly, the experiment used a 2 × 2 factorial design where one factor was *search type* (feature, conjunction) and the other factor was cost (low, high). The search control conditions were: feature-low, feature-high, conjunction-low, and conjunction-high. The control trials were designed to assess differences in baseline costs for search display conditions (i.e., in isolation for other search displays), whereas the test trials were designed to assess differences in search preference (i.e., in the presence of alternative search displays), hence the different factors for control and test trials.

### 2.4. Procedure and analysis

Each participant underwent the following procedure. First, instructions were given on the task procedure. Participants were instructed to identify notch position of the target in either the left or right screen. Participants were told that notch position was the same for both targets. A practice session was then administered to ensure that they understood the requirements. The practice session consisted of one block of eight control trials followed by one block of eight test trials. After confirming that the participants understood the task, they proceeded to the main task.

The main task consisted of eight control and eight test blocks of trials, in random order. Each block consisted of 32 trials (four conditions by eight repetitions). Hence, each participant received a total of 512 trials. For half of the low cost feature search trials there were seven blue and eight purple distractors, and for the other half of those trials there were eight blue and seven purple distractors. The same distribution of distractor colors applied to the high cost conjunction search trials. The order of trial conditions was randomized within each block. Feature and conjunction search sets were randomly assigned to the left or right hemifields, and counterbalanced. For test trials, the left-right assignment of search sets was either feature-conjunction, or conjunction-feature. Assignment of objects to grid cells in each search field was random, except that targets were restricted to cells in the outer halves (three columns) of each grid. At the start of each block, an information screen with a sample display was presented to identify the subsequent trials for the current block as being either all one-set (control) or all two-set (test) trials. Blocks were self-paced: pressing the “space” key started the sequence of trials for that block.

Each trial consisted of three phases: (1) the *fixation* phase (2,000 ms), to indicate the start of a new trial, (2) the *search* phase, and (3) the *end* phase (500 ms), to indicate the end of the current trial, in that order. During the fixation phase, a small white dot appeared at the center of the screen for the 0–400 ms and 800–2,000 ms intervals. The search phase consisted on one (control) or two (test) sets of display objects, and the fixation point. During this phase, participants were required to press the “F” or “J” keys to indicate that the notch was located at (respectively) the bottom or top of the target object. A trial was regarded as a correct response trial if the participant pressed the key corresponding to the notch location by the end of the search phase, otherwise it was regarded as an error trial. Thus, correct response rate is the proportion of correct response trials to total number of trials for the specified condition. The search phase ended upon pressing either key. The end phase consisted of a blank screen. Accuracy and speed of response were emphasized. Response choices, response times, and eye movements were recorded. The total time required to complete the experiment was about 1 h.

Two-way analyses of variance (ANOVAs) with search type and search cost as factors for control trials, and feature cost and conjunction cost as factors for test trials were conducted to assess differences in mean error rates and response times. Analysis of response times was conducted on error-free trials. The following processing steps were applied to the EOG data.

1. Data were band-pass filtered at 0.01–30 Hz.
2. Correction for voltage changes in the electrodes assigned to detect horizontal eye movement were derived from the voltages of the electrodes assigned to detect vertical eye movement using Gratton's method (Gratton et al., 1983).
3. Trials deemed as response time outliers by the recursive rejection method (Selst and Jolicoeur, 1994) calculated from all (correct and incorrect response) trials were removed from subsequent analysis.
4. Mean voltage amplitudes were calculated for the 0–100 ms interval that preceded response key onset.
5. Gaze direction was determined by the sign of the mean voltage amplitude relative to the pre-stimulus baseline: negative voltage implied left hemifield; positive voltage implied right hemifield.

Two-way ANOVAs were used to assess mean gaze direction rate relative to the location of the feature search set for test trials. Two-tail *t*-tests against chance level (0.5) were used for control trials, because these trials only contained objects in one hemifield. So, a gaze rate of 1 indicates that gaze direction was always to the hemifield that contained the feature search set, regardless of whether that set was contained in the left or right hemifield; a gaze rate of 0 indicates that gaze direction was always to the hemifield that contained the conjunction search set, regardless of whether that set was in the left or right hemifield.

## 3. Results and discussion

### 3.1. Behavioral data

Behavioral and EOG data were conducted on all trials not rejected as outliers. The mean rejection rate across participants was 0.029; the minimum and maximum within-participant rates were 0.003 and 0.102.

#### 3.1.1. Control trials

For correct response rates, there were no significant main effects. Correct response rates were above 0.95 in all conditions. For response times, there was a significant main effect of cost, *F*_(1, 22)_ = 29.51, *p* < 0.001, but not search, *F*_(1, 22)_ = 0.01, *p* = 0.9. There was no significant interaction. Participants were faster to respond in the low cost condition than the high cost condition.

#### 3.1.2. Test trials

**Figure 2:**
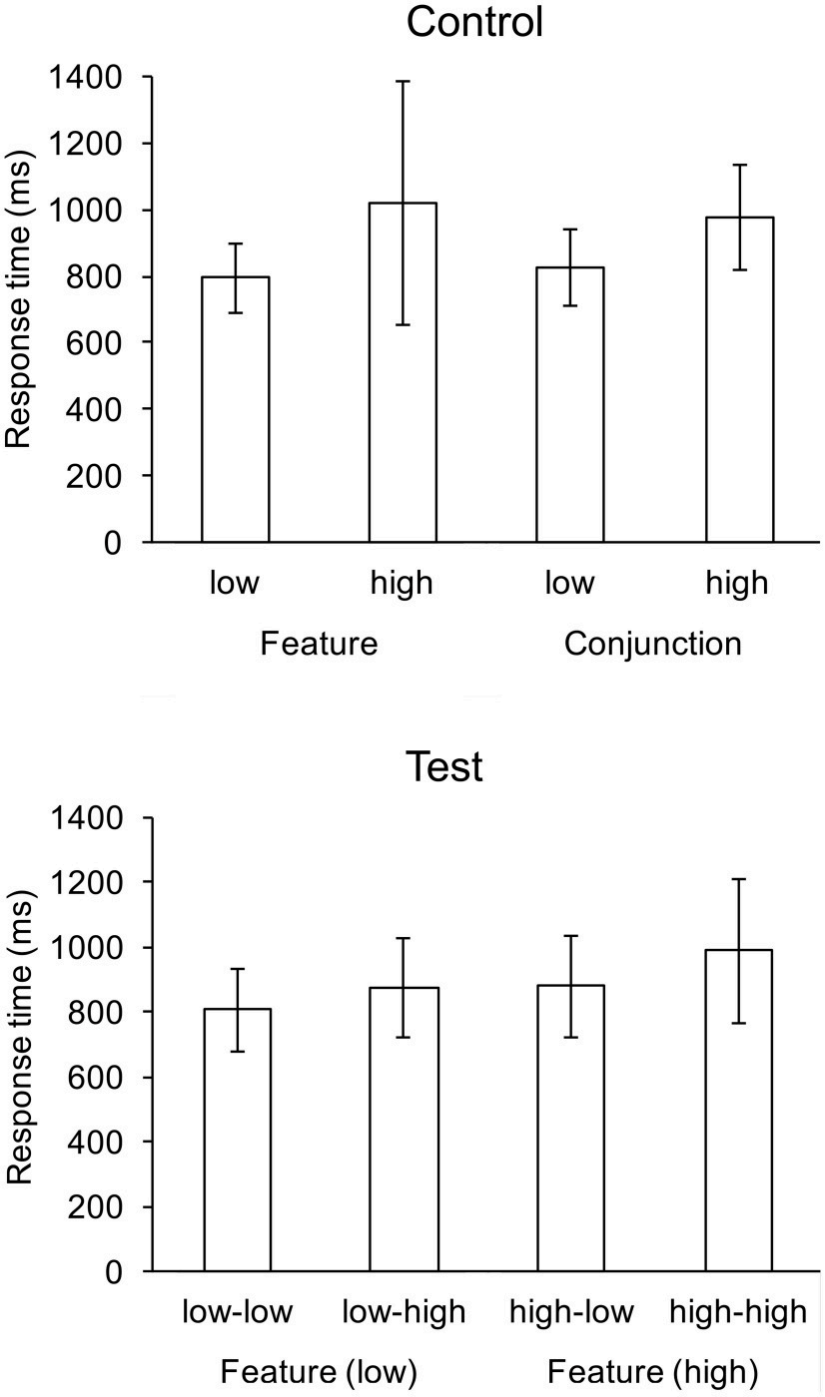
Mean response times (ms) for control and test conditions. Whiskers indicate one standard deviation.

There were no main effects for response rates. Response rates for all conditions were above 0.95. For response times, there were significant main effects of feature search cost, *F*_(1, 22)_ = 61.39, *p* < 0.001, and conjunction search cost, *F*_(1, 22)_ = 72.65, *p* < 0.001, and there was a significant interaction, *F*_(1, 22)_ = 4.39, *p* < 0.05. Response was fastest in the low-low condition (807 ms), and slowest in the high-high condition (989 ms). The interaction was influenced by the greater difference in mean response times between the high-high and high-low conditions (109 ms) than between the low-high and low-low conditions (67 ms). The difference between high-high and high-low conditions did not reach significance; likewise, the difference between low-high and low-low conditions. Mean response times are shown in Figure 2.

To compare display search in isolation with display search in the context of an alternative search display, two-tailed *t*-tests were performed for all pairwise combinations of control and test conditions, e.g., feature-low with low-low Table 1. Search in the feature-low and conjunction-low conditions were significantly faster than search in the low-high, high-low and high-high conditions. Conversely, search in the feature-high and conjunction-high conditions were significantly slower than search in the low-low, low-high and high-high conditions.

**Table 1:** Response time contrasts of control (row labels) vs. test (column labels) trial conditions: *t*-scores (*p*-values).

	**Low-low**	**Low-high**	**High-low**	**High-high**
Feature-low	−1.02 (0.319)	−4.88 (0.001)	−5.30 (0.001)	−6.54 (0.001)
Feature-high	3.60 (0.002)	2.64 (0.015)	2.77 (0.011)	0.64 (0.526)
Conjunction-low	1.96 (0.062)	−3.04 (0.006)	−3.33 (0.003)	−5.87 (0.001)
Conjunction-high	11.33 (0.001)	6.43 (0.001)	6.37 (0.001)	−0.40 (0.696)

### 3.2. EOG data

#### 3.2.1. Control trials

For each of the four control conditions, the mean gaze rate to the search field was at least 0.98, which was significantly greatly than chance (0.5): *t*_(22)_ = 153.92, *p* < 0.001 (low feature), *t*_(22)_ = 97.11, *p* < 0.001 (high feature), *t*_(22)_ = 121.79, *p* < 0.001 (low conjunction), and *t*_(22)_ = 185.72, *p* < 0.001 (high conjunction).

#### 3.2.2. Test trials

**Figure 3:**
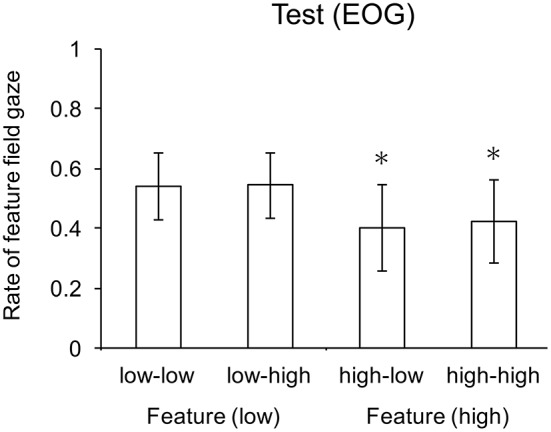
Mean gaze rates to the feature search field. Whiskers indicate one standard deviation, and ^*^ indicates means significantly lower than chance.

There was a significant main effect of feature search on gaze rate, *F*_(1, 22)_ = 8.43, *p* < 0.01, but not conjunction search cost, *F*_(1, 22)_ = 0.81, *p* = 0.38. There was no significant interaction. Participants more often gazed to the hemifield containing the feature search set when feature search cost was low than when features search cost was high. Two-tail *t*-tests revealed that average gaze rate for the two conjunction search cost conditions (i.e., high-low and high-high) were significantly below the 0.5 chance rate (*p* < 0.01). Separately, the gaze rates for these two conditions were also significant: high-low (*p* < 0.005) and high-high (*p* < 0.02) conditions. Gaze rate for the two feature search cost conditions (i.e., low-low and low-high) were not significantly above chance level (*p* = 0.076); specifically, in the low-low condition (*p* = 0.109) and the low-high condition (*p* = 0.071). Mean gaze rates to the feature search field are shown in Figure 3.

The data provide support for the context least-cost hypothesis, but not the independent or absolute hypotheses. Response times for the control conditions provide the baseline cost for each combination of search and cost conditions. Eye movement data for control conditions indicates that participants overtly attend to the hemifield containing the search field, which supports this measure as an indicator of route selection for the test conditions. The significant difference in the cost factor for both feature and conjunction search conditions shows that discriminability (feature) and dimensionality (conjunction) were effective in manipulating search difficulty/cost. The lack of significant difference between search (feature vs. conjunction) at each level of cost (low and high) shows that these conditions were comparable: search in the feature-low condition was as costly as search in the conjunction-low condition, and search in the feature-high condition was a costly as search in the conjunction-high condition. These equivalences indicate that alternation is not a simple consequence of feature vs. conjunction search, but instead influenced by (meta-)cognitive processes that evaluate the relative costs of each computational path. So, we have a basis for considering the contextualized least cost hypothesis. Next, we assess each hypothesis in the light of test trial (behavioral and EOG) data, when these conditions were opposed.

### 3.3. Independent and absolute hypotheses

Although the response time data is consistent with the independent hypotheses, there is no support for this hypothesis when considered in conjunction with the EOG data. The independent hypothesis is consistent with the apparent averaging of the response times for the control conditions that constitute the opposed conditions in the test trials. That is, the response time for the low-low condition (~800 ms) was about the same as the response times for the feature-low and conjunction-low conditions, the high-high condition (~1,000 ms) was about the same as for feature-high and conjunction-high conditions, and the low-high and high-low conditions were between their respective constituent controls (see Table 1 for detailed comparisons). Thus, simply attending to the same hemifield, or randomly to either hemifield would yield an average of the alternatives as given by the control trials. However, the independent hypothesis predicts no difference in gaze rate to the feature search field, due to the random assignment of search field to left/right display hemifield, so the significant difference in feature cost (low vs. high) on gaze rate counts against this hypothesis. Hence, the EOG data rule out the independent hypothesis.

The response time data together with the EOG data do not support the absolute least-cost hypothesis. This hypothesis predicts response times on test trials as the minimums of the responses times for control trials in the corresponding alternative conditions: response times in the low-low, low-high, and high-low conditions as comparable (because participants utilized the low cost alternative in each case) and less than the high-high condition. Although response times for the low-high and high-low conditions were comparable, both were longer than the low-low condition. One could argue that this difference results from additional time needed to evaluate relative costs when one alternative is a high cost condition. However, this situation still implies a significant difference in gaze direction: participants should search more often in the low cost alternative, even though more time is taken to make that decision. Yet, the EOG data indicating no significant difference in gaze direction between high-low and high-high conditions do not support this argument. Thus, taken together, the response time and EOG data do not support the absolute hypothesis.

### 3.4. Context least-cost hypothesis: assessing relative cost

Lack of empirical support for both independent and absolute hypotheses is implicit support for the context least-cost hypothesis, in the sense that some other factor (or combination of other factors) is involved. The response time data indicate that participants were only partly sensitive to the relative costs of the opposing search conditions: recall that search time in the low-high and high-low conditions were comparable, but longer than the low-low condition. We now turn to possible context-sensitive factors.

The gaze rate data suggest that the context sensitivity lies with the relative difficulty of determining feature vs. conjunction search cost: participants more readily distinguish low vs. high feature search cost than low vs. high conjunction search cost. One reason may lie with the difficulty in determining the dimensionality of the conjunction search field compared with determining the discriminability of the colors in the feature search field. In feature conditions, the color dimension varies categorically in the low cost condition (i.e., red vs. non-red) but metrically in the high cost condition (red vs. orange vs. beige), whereas in the conjunction search conditions, color varies categorically in both low and high cost conditions. So, choice based on feature cost offers a compromise between the cost of assessing search conditions and the consequences of assessing those conditions incorrectly. For the low-low and high-high conditions, a choice based on feature condition offers no performance disadvantage, since the alternatives have equal base costs (according to the control response times). For the high-low condition, feature-based choice was serendipitously advantageous, since search in the conjunction-low condition was faster than in the feature-high condition. So, feature condition assessment explains (or, is at least consistent with) the EOG data for those three conditions. However, the lack of a significant gaze effect for the low-high condition, in contrast to the high-low condition does not provide support for this explanation, although this difference may simply reflect a lack of statistical power given the trend in gaze rate to the feature search field when the data were collapsed over low-low and low-high conditions (*p* = 0.076). Consequently, we conducted a follow-up experiment to test the cost of computing least-cost hypothesis, discussed next.

## 4. Cost of computing least-cost hypothesis

The purpose of this follow-up experiment was to test the cost of computing least-cost hypothesis, by opposing low vs. high feature search in one condition, and low vs. high conjunction search in the other condition. The cost of computing least cost hypothesis predicts a significant difference in gaze rate to the low as opposed to the high feature search field, but not to the low as opposed to the high conjunction search field. In addition to testing this hypothesis, we also replicated the experiment reported in section 3. Thus, each participant undertook both replication and follow-up experiments. The order of the two experiments was randomized. This section describes the design of the follow-up experiment only.

### 4.1. Participants

Twenty-one adults (19–30 years of age, 16 males, 20 right handed) were recruited for the follow-up experiment and were paid for their participation. All participants had normal or corrected-to-normal eyesight. None of the participants for the follow-up experiment participated in the first experiment.

### 4.2. Apparatus and stimuli

The apparatus and stimuli used for the follow-up experiment were the same as for the previous experiment.

### 4.3. Conditions

The follow-up experiment, concerned with testing the cost of computing least-cost hypothesis, involved a one-factor (two-level) design. The factor is search type and the levels were feature and conjunction. The feature condition opposed a low feature search field against a high feature search field, and the conjunction condition opposed a low conjunction search field against a high conjunction search field. Hence, the follow-up experiment consisted of just two trial types, where (1) a feature low cost display was paired with a feature high cost display, and (2) a conjunction low cost display was paired with a conjunction high cost display. All other conditions for the follow-up experiment were the same as before.

### 4.4. Procedure and analysis

The procedures and analyses for the follow-up experiment were the same as for the previous experiment. Accordingly, low and high search fields were randomly allocated to the left and right display hemifields.

### 4.5. Results and discussion

#### 4.5.1. Replication

**Figure 4:**
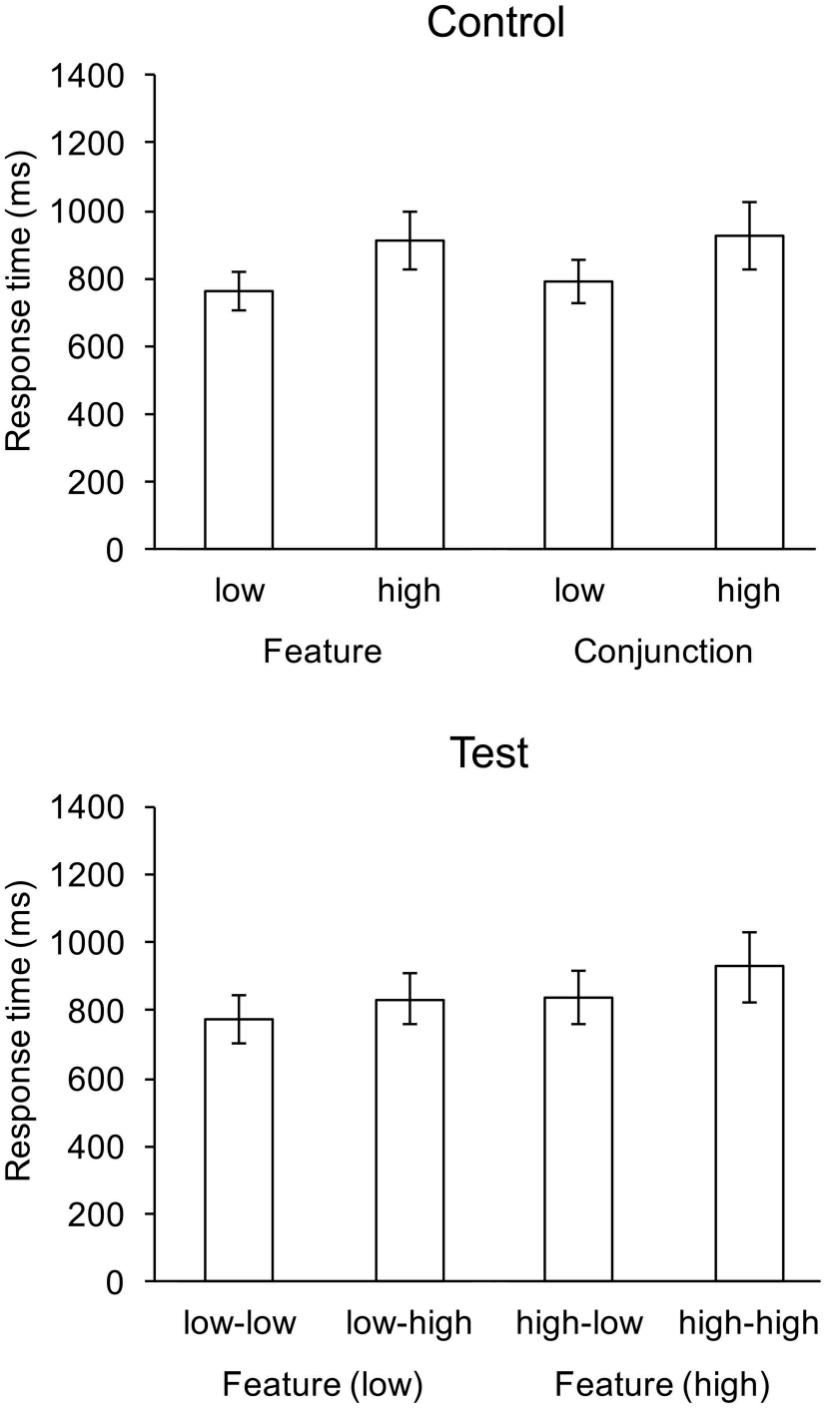
Mean response times (ms) for control and test conditions of the replication experiment. Whiskers indicate one standard deviation.

The ANOVA for response times on test trials replicated the effects observed on test trials for the previous experiment. For control trials, the mean gaze rate to the search field for each of the four control conditions was at least 0.97, which was significantly greatly than the 0.5 chance rate: *t*_(20)_ = 17.558, *p* < 0.001 (low feature), *t*_(20)_ = 30.403, *p* < 0.001 (high feature), *t*_(20)_ = 31.969, *p* < 0.001 (low conjunction), and *t*_(20)_ = 37.282, *p* < 0.001 (high conjunction). For test trials, there were significant main effects of features search cost, *F*_(1, 20)_ = 147.6, *p* < 0.001, and conjunction search cost, *F*_(1, 20)_ = 151.17, *p* < 0.001, and there was a significant interaction, *F*_(1, 22)_ = 5.91, *p* < 0.05. Response was fastest in the low-low condition, and slowest in the high-high condition. The ANOVA for control trials replicated the effect of cost on response times for the previous experiment, *F*_(1, 22)_ = 163.66, *p* < 0.001, but there was also an effect of search on response times that was not observed for the previous experiment, *F*_(1, 22)_ = 11.62, *p* < 0.01, feature search was faster the conjunction search. With regard to EOG, there was a significant main effect of feature cost on gaze rate, *F*_(1, 22)_ = 14.75, *p* < 0.001. The main effect of conjunction cost on gaze rate was marginal *F*_(1, 22)_ = 4.21, *p* = 0.054. The interaction was not significant. Mean response times are shown in Figure 4.

**Figure 5:**
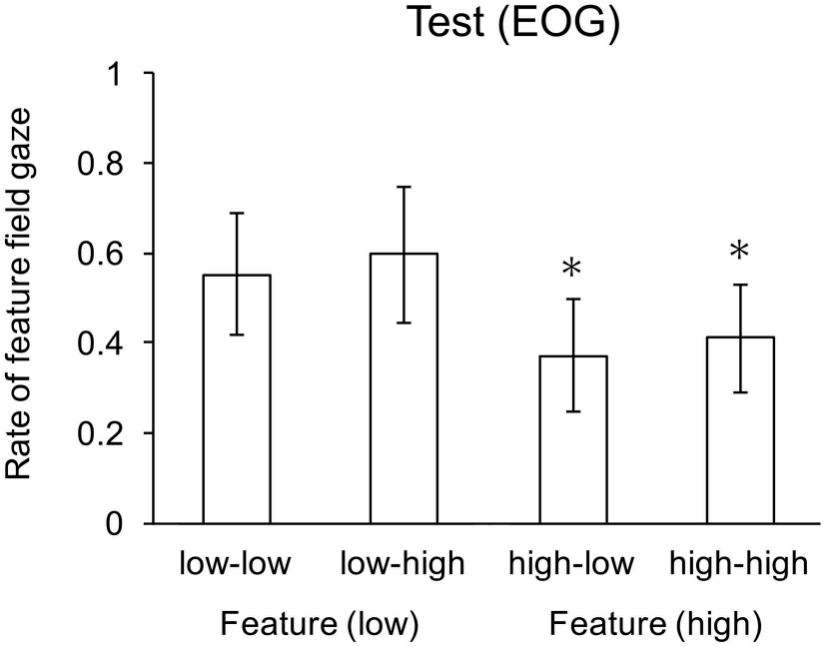
Mean gaze rates to the feature search field. Whiskers indicate one standard deviation, and ^*^ indicates means significantly lower than chance.

Two-tailed *t*-tests revealed that the mean saccade rate in the (feature-conjunction) high-low and high-high conditions were significantly lower that chance. Mean rates for each condition are shown in Figure 5. As with the previous experiment, there were no significant effects for response rates, which were near ceiling (greater than 0.95) for all conditions.

#### 4.5.2. Cost of determining least-cost (follow-up experiment)

The mean response time (standard deviation) for the feature low-high condition of 829 ms (70 ms) was shorter than the mean response time for the conjunction low-high condition 845 ms (97 ms), although this difference did not reach significance, *t*_(20)_ = −1.85, *p* = 0.079 (two-tailed).

**Figure 6:**
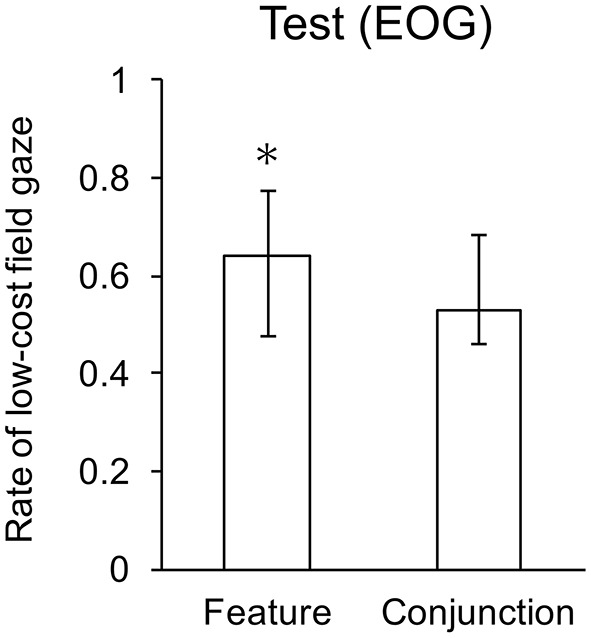
Mean gaze rates to the low search cost field. Whiskers indicate one standard deviation, and ^*^ indicates means significantly lower than chance.

Analysis of EOG for low vs. high difficulty in feature and conjunction search revealed a significant effect of search type, *F*_(1, 22)_ = 9.46, *p* < 0.01. Two-tailed *t*-tests revealed that the mean gaze rate was significantly greater than chance in the feature condition, but not the conjunction condition. Mean gaze rates are shown in Figure 6.

The data confirm the cost of computing least-cost hypothesis: participants assess the relative difficulty of low vs. high feature conditions, but not low vs. high conjunction conditions. Although the response time difference did not reach significance, the trend toward faster response in the feature low-high condition is consistent with the difference in gaze direction, since participants conducted significantly more searches in the lower cost search display for the feature than for the conjunction alternatives.

## 5. General discussion

The data support a contextualized version of the least-cost principle in that the cost of computing least-cost also influences display selection, where cost was based on the response times for search when only one display set was presented (i.e., the no-alternative display/route control trials). Participants primarily based their decision on feature search cost, as suggested by the response time and gaze direction data from the first experiment—sensitivity to conjunction-search cost implies that participants should search for the target more often in the conjunction display for the high-low than high-high condition, which would be at chance level. However, no significant difference was observed. The difficulty of assessing conjunction search cost was confirmed in the follow-up experiment, which revealed a clear low cost preference when the alternatives were low or high cost feature search, but not when the alternatives were low or high cost conjunction search. Thus, participants assessed alternatives, but only when the cost of that assessment was relatively low.

Note that the cost of assessing relative costs must itself be relatively cheap, or automatically invoked, otherwise there is the potential for infinite regress: if this (second-order) assessment of cost itself requires a cost assessment, then there must be a third-order cost assessment, and so on. One way to short-circuit this infinite regress is to enforce a time-limit on the decision process. Suppose the decision is govern by a race to some criterion threshold (see Ratcliff and Smith, 2004). By imposing a time constraint, choice defaults to the leading alternative, thus circumventing an infinite regress of cost assessments.

One caveat to the current study concerns the relationship between dual-display as distinguished task conditions and dual-route as distinguished cognitive processes. In our task, the two displays yielded the same responses. So, cognitive functions cannot be distinguished *extensionally*: by different input-output relations; rather, they can only be distinguished *intensionally*: e.g., by differences in constituent functions—functional equation *f* ◦ *g* = *h* indicates extensional equality in that both sides specify the same input-output relation, but not intensional equality because the left side is composed of two functions (*f* and *g*) and the right side only one (*h*). A direct way of identifying cognitive dual-routes is to contrast processes that make contradictory behavioral (response) predictions (e.g., Sternberg and McClelland, 2012). However, our interest here is in complementary cognitive processes as introduced earlier. And so, although response time and gaze rate differences suggest alternative cognitive processes, further work is needed to tease apart putative differences in constituent processes, which we discuss next.

Clearly, participants are partly sensitive to the relative cost of search. Less straightforward is assessing whether the alternative displays necessitate different search processes: exercise a dual-route processing strategy. Recall (Introduction) the distinction between pluralistic (hybrid) vs. monolithic theories. We suppose that one could argue for a single search process to account for feature and conjunction search (monolithic theory), given that the baseline response times (control trials) were comparable for the same level of difficulty. Presumably, for example, such an argument could proceed by assuming a comparable similarity metric for items in feature and conjunction displays, where comparable measures of target-distractor and distractor-distractor similarity influence search efficiency akin to, say, the Guided Search Model (Wolfe et al., 1989). On the other hand, a (single) mechanism that accounts for assessment of feature search difficulty may well be necessary, but not sufficient to explain the inability to assess the difficulty of conjunction search. One can always assume a single function that accommodates all conditions by including additional parameters or assumptions, but the important question is whether such additions are *ad-hoc* to the monolithic theory (Aizawa, 2003).

Toward this end, we outline some category theory (Mac Lane, 1998) connections to help push forward this work. Universal constructions (e.g., categorical products) are central to an explanation of systematicity (Phillips and Wilson, 2010) (See also Phillips et al., 2009 for categorical products as an explanatory component of cognitive developmental capacity). Products and pairs of functions are equivalent: a single function over a set of pairs compares with a pair of functions each over a corresponding set of singletons. Such equivalences induce two equal but distinct paths, typically expressed by so-called *commutative diagrams*. One can interpret conjunction search as involving a (binary or ternary) product and feature search as involving a non-product (or, trivial unary product). So, (double/triple) conjunction search involves a single search along multiple (two/three) feature dimensions, or multiple searches each along a feature dimension. Visual conjunction search reportedly involves greater EEG synchrony with product arity (Phillips et al., 2012), hence the category theoretical motivation for the current study (Systematicity in this situation is having, e.g., the capacity to search for a blue triangle among blue squares and red triangles whenever having the capacity to search for a red square among red triangles and blue squares, assuming the ability to recognize the constituent color and shape features). The general methodological challenge is to develop further empirical tests for the implied equivalent computational paths.

## Author contributions

All authors listed, have made substantial, direct, and intellectual contribution to the work, and approved it for publication. SP, YT, and FS designed the experiment, analyzed the data, and wrote the manuscript. FS implemented the experiment and collected the data.

### Conflict of interest statement

The authors declare that the research was conducted in the absence of any commercial or financial relationships that could be construed as a potential conflict of interest.

## References

[B1] Aizawa K. (2003). The Systematicity Arguments, Studies in Mind and Brain. New York, NY: Kluwer Academic.

[B2] Buschman T. J. Miller E. K. (2007). Top-down versus bottom-up control of attention in the prefrontal and posterior parietal cortices. Science 315, 1860–1862. 10.1126/science.1138071 17395832

[B3] Chater N. Oaksford M. (eds.). (2008). The Probabilistic Mind: Prospects for Bayesian Cognitive Science. New York, NY: Oxford University Press.

[B4] Christoff K. Prabhakaran V. Dorfman J. Kroger K. J. Zhao Z. Holyoak K. J. (2001). Rostral prefrontal cortex involvement in relational processing during reasoning. Neuroimage 14, 1136–1149. 10.1006/nimg.2001.0922 11697945

[B5] Evans J. S. B. T. Stanovich K. E. (2013). Dual-process theories of higher cognition: advancing the debate. Perspect. Psychol. Sci. 8, 223–241. 10.1177/1745691612460685 26172965

[B6] Everett D. L. (2016). An evaluation of universal grammar and the phonological mind. Front. Psychol. 7:15. 10.3389/fpsyg.2016.00015 26903889 PMC4744836

[B7] Fodor J. A. McLaughlin B. P. (1990). Connectionism and the problem of systematicity: why Smolensky's solution doesn't work. Cognition 35, 183–204. 10.1016/0010-0277(90)90014-B 2354612

[B8] Fodor J. A. Pylyshyn Z. W. (1988). Connectionism and cognitive architecture: a critical analysis. Cognition 28, 3–71. 10.1016/0010-0277(88)90031-5 2450716

[B9] Gratton G. Coles M. G. Donchin E. (1983). A new method for off-line removal of ocular artifact. Electroencephalogra. Clin. Neurophysiol. 55, 468–484. 10.1016/0013-4694(83)90135-9 6187540

[B10] Halford G. S. Wilson W. H. Andrews G. Phillips S. (2014). Categorizing Cognition: Toward Conceptual Coherence in the Foundations of Psychology. Cambridge, MA: MIT Press.

[B11] Johnson K. (2004). On the systematicity of language and thought. J. Philos. 101, 111–139. 10.5840/jphil2004101321

[B12] Johnson-Laird P. N. Khemlani S. S. Goodwin G. P. (2015). Logic, probability, and human reasoning. Trends Cogn. Sci. 19, 201–214. 10.1016/j.tics.2015.02.006 25770779

[B13] Kahneman D. (2011). Thinking, Fast and Slow. New York, NY: Farrar, Straus and Giroux.

[B14] Lloyd M. E. Doydum A. O. Newcombe N. S. (2009). Memory binding in early childhood: evidence for a retrieval deficit. Child Dev. 80, 1321–1328. 10.1111/j.1467-8624.2009.01353.x 19765002

[B15] Mac Lane S. (1998). Categories for the Working Mathematician, Graduate Texts in Mathematics, 2nd Edn. New York, NY: Springer.

[B16] Niklasson L. van Gelder T. (1994). On being systematically connectionist. Mind Lang. 9, 288–302. 10.1111/j.1468-0017.1994.tb00227.x

[B17] Phillips S. Takeda Y. Singh A. (2012). Visual feature integration indicated by phase-locked frontal-parietal EEG signals. PLoS ONE 7:e32502. 10.1371/journal.pone.0032502 22427847 PMC3302878

[B18] Phillips S. Takeda Y. Sugimoto F. (2016). Why are there failures of systematicity? The empirical costs and benefits of inducing universal constructions. Front. Psychol. 7:1310. 10.3389/fpsyg.2016.01310 27630596 PMC5005328

[B19] Phillips S. Wilson W. H. (2010). Categorial compositionality: a category theory explanation for the systematicity of human cognition. PLoS Comput. Biol. 6:e1000858. 10.1371/journal.pcbi.1000858 20661306 PMC2908697

[B20] Phillips S. Wilson W. H. Halford G. S. (2009). What do transitive inference and class inclusion have in common? Categorical (co)products and cognitive development. PLoS Comput. Biol. 5:e1000599. 10.1371/journal.pcbi.1000599 20011111 PMC2781167

[B21] Ratcliff R. Smith P. L. (2004). A comparison of sequential sampling models for two-choice reaction time. Psychol. Rev. 111, 333–367. 10.1037/0033-295X.111.2.333 15065913 PMC1440925

[B22] Selst M. V. Jolicoeur P. (1994). A solution to the effect of sample size on outlier elimination. Q. J. Exp. Psychol. 47A, 631–650. 10.1080/14640749408401131

[B23] Sternberg D. A. McClelland J. L. (2012). Two mechanisms of human contingency learning. Psychol. Sci. 23, 59–68. 10.1177/0956797611429577 22198929

[B24] Thomas N. J. T. (2016). Mental imagery in The Stanford Encyclopedia of Philosophy, Winter 2016 Edn, ed Zelta E. N. (Stanford, CA: Stanford University). Available online at: https://plato.stanford.edu/archives/win2016/entries/mental-imagery/

[B25] Treisman A. M. Gelade G. (1980). A feature-integration theory of attention. Cogn. Psychol. 12, 97–113. 10.1016/0010-0285(80)90005-5 7351125

[B26] Waltz J. A. Knowlton B. J. Holyoak K. J. Boone K. B. Mishkin F. S. Santoa M. D. M. (1999). A system for relational reasoning in human prefrontal cortex. Psychol. Sci. 10, 119–125. 10.1111/1467-9280.00118

[B27] Watumull J. Hauser M. D. Roberts I. G. Hornstein N. (2014). On recursion. Front. Psychol. 4:1017. 10.3389/fpsyg.2013.01017 24409164 PMC3884515

[B28] Wolfe J. M. Cave K. R. Franzel S. L. (1989). Guided search: an alternative to the feature integration model for visual search. J. Exp. Psychol. Hum. Percept. Perform. 15, 419–433. 10.1037/0096-1523.15.3.419 2527952

